# Break Down the Silos: A Conceptual Framework on Multisectoral Approaches to the Prevention and Control of Vector-Borne Diseases

**DOI:** 10.1093/infdis/jiaa344

**Published:** 2020-10-29

**Authors:** Qingxia Zhong, Florence Fouque

**Affiliations:** UNICEF/UNDP/World Bank/WHO Special Programme for Research and Training in Tropical Diseases (TDR), Geneva, Switzerland

**Keywords:** multisectoral approach, vector-borne diseases (VBDs), theoretical framework

## Abstract

The need for multisectoral approaches (MSAs) in prevention and control of vector-borne diseases (VBDs) has been identified. VBD programs often involve collaborations between health and nonhealth sectors; however, a systematic framework describing the process, requirements, challenges, and benefits of MSAs has been missing. A recent guidance document from UNICEF/UNDP/World Bank/WHO Special Programme for Research and Training in Tropical Diseases provides a framework to implement MSAs for prevention and control of VBDs. This article gives an introduction to the guidance document and describes the conceptual framework and coordination process. The next steps will be to test the framework through implementation research in specific VBD cases studies. The guidance document will thus be refined based on iterative and comprehensive monitoring and evaluation systems to assess the performance and impact of MSAs. The advocacy for MSA and necessary capacity building will be integrated into the testing of the framework.

Despite the global effort to combat vector-borne diseases (VBDs), more than 80% of the world population is still exposed to at least 1 major VBD [1]. An estimated loss of 50 million disability-adjusted life years was attributed to 4 major diseases transmitted by mosquitoes (malaria, dengue, lymphatic filariasis, and yellow fever) in 2017 [2]. The prevention and control of VBDs is usually under the health sector. One such example is the malaria elimination program launched by the Ministry of Health of Indonesia in Sabang Municipality through mainly vector assessment, map and database development on malaria, stratification and mapping residual foci of malaria transmission, and mass blood screening [3]. However, some very efficient interventions cannot be carried out by the health sector alone and need input from other sectors, such as water and sanitation, agriculture, housing, or education. Other emerging issues, such as insecticide resistance, urbanization, climate change, and population displacement, further exacerbate the control challenge. Therefore, a multisectoral approach (MSA) is required to sustain the control of VBDs by harnessing the strengths of multiple sectors, taking into account all the relevant determinants.

## THE DEFINITION OF A MULTISECTORAL APPROACH

Multisectoral and intersectoral approaches are neither new nor reemerging trends in the field of public health. A simple search in PubMed of these 2 words in the titles and abstracts identified the first documented studies in the 1970s, notably on health self-reliance in Tanzania [4], the national health program and primary health care in Sudan [5], and integrated population, nutrition, and food programs in Asia and the Pacific region [6]. At about the same time, the Declaration of Alma-Ata in 1978 formally introduced the concept that primary health care should involve all related sectors such as (but not limited to) agriculture, animal husbandry, and education [7]. This approach is relevant to all health outcomes including VBDs. A brief look into the history of vector control revealed many examples involving collaboration with nonhealth sectors [8]. The emergence, transmission, and distribution of VBDs are linked to a complex set of determinants, which extend beyond the capacities of the health sector. Therefore, including relevant nonhealth sectors is a necessity. In 2004, the World Health Organization (WHO) developed the *Global Strategic Framework for Integrated Vector Management*, which emphasized the need to collaborate with other sectors [9]. In the WHO’s *Global Vector Control Response 2017–2030*, “strengthen inter- and intra-sectoral action and collaboration” is recommended as 1 of the 4 pillars of action [10], and in the WHO *Thirteenth General Program of Work 2019–2023*, the need for strengthening MSAs is highlighted to address all the determinants of health and to achieve a paradigm shift [11].

The terms “multisectoral” and “intersectoral” have often been used interchangeably; however, semantically “multi-” focuses on “more than one, several, many,” and “inter-” refers to the interaction “between, among, amid” several actors [12]. As such, in this article as well as in the guidance document developed by United Nations Children's Fund (UNICEF)/ United Nations Development Programme (UNDP)/World Bank/WHO Special Programme for Research and Training in Tropical Diseases (TDR) [13], “multisectoral” is favored to emphasize the importance of going beyond the health sector alone, and to engage several (multi) relevant sectors. Several documents have been developed to discuss this concept. Consolidating from the *Intersectoral Action for Health* [14, 15], the *Multisectoral Action Framework for Malaria* [16], *Global Strategic Framework on Integrated Vector Management* [9], the *Action and Investment to Defeat Malaria 2016–2030* [17], and United States Agency for International Development’s Health Policy Project [18], an MSA can be defined as [13]: A recognized relationship between part or parts of the health sector with part or parts of several other sectors, including governmental sectors, public and private institutions and organizations, NGOs and others, which has been formed to take action on an issue to achieve health outcomes (or intermediate health outcomes) in a way that is more effective, efficient or sustainable than could be achieved by the health sector alone.

## THE ADVANTAGES AND BENEFITS OF AN MSA

**Figure 1.**
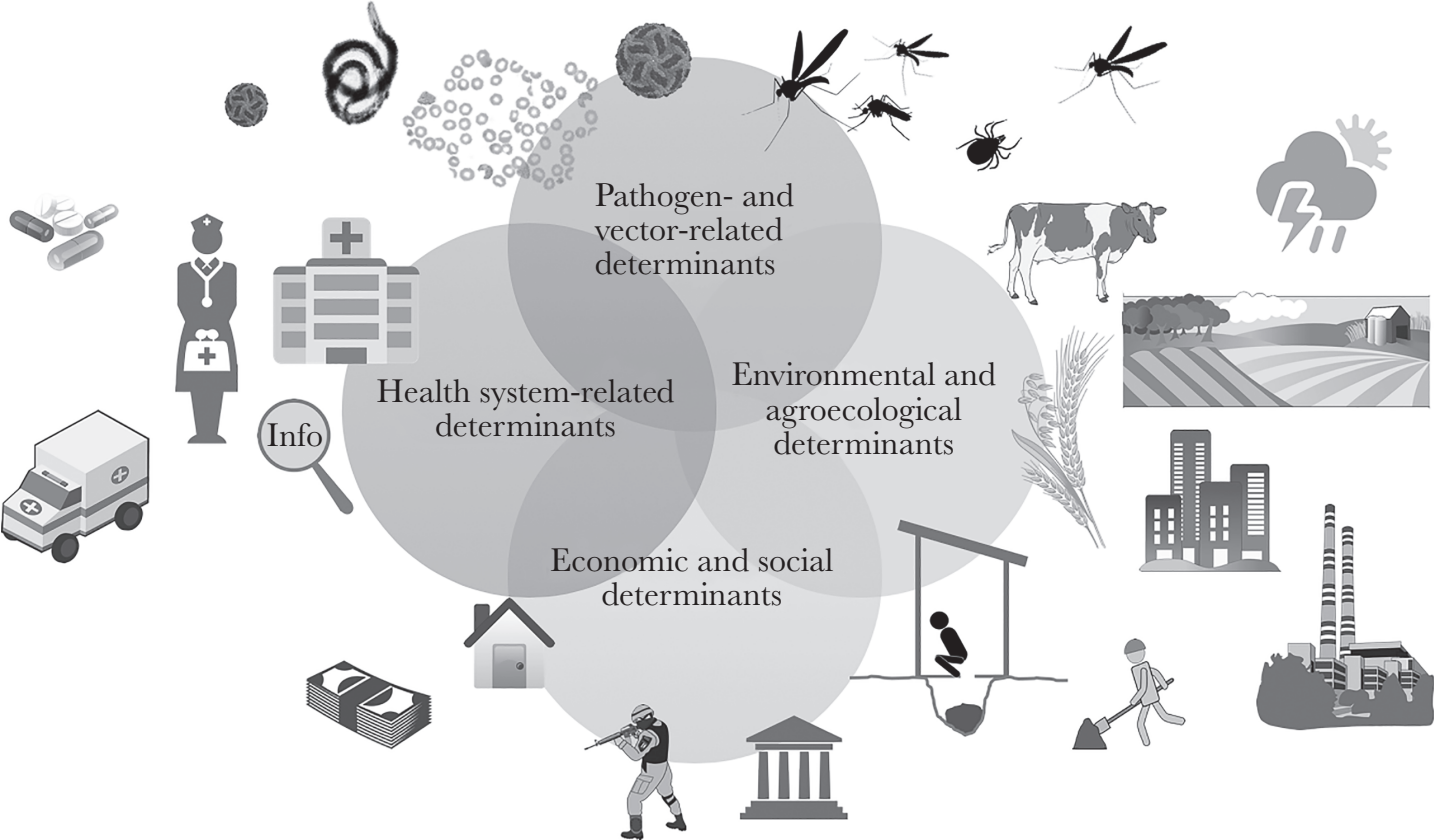
Determinants of vector-borne diseases. Adapted from the guidance document [13].

As mentioned above, the determinants of VBDs span across disciplines and can be grouped into 4 broad categories (Figure 1): (1) pathogen- and vector-related determinants, such as vector host seeking and biting behavior, and pathogen mutation; (2) environmental and ecological determinants, such as vegetation cover and the type of water body; (3) economic and social determinants, such as human behavior, agricultural practices, poverty, and growth of urban slums; and (4) health system-related determinants, such as the access to adequate health care facilities. These determinants are intertwined and partially overlapping. Other emerging global issues, such as climate change and population displacement, further complicate prevention and control strategies.

MSA can strengthen health programs for VBDs prevention and control by achieving the following: (1) leveraging knowledge, expertise, and reach (geographically, demographically, and administratively) of multiple sectors; (2) increasing synergy and coherence in each sector’s policies and activities with regard to VBDs; (3) strengthening capacity in all relevant sectors and empowering community; (4) fostering resource sharing, optimizing resource use, and maximizing impact; (5) tackling all relevant determinants of VBDs; and (6) facilitating scaling-up and increasing sustainability both institutionally and financially.

## SOME THEORETICAL CONCEPTS ON HOW TO IMPLEMENT AN MSA

TDR’s newly developed guidance document [13] aims to help member states and any other interested body (institution, organization, private sector, etc.) with some theoretical concepts extracted from evidence and experience on the best way to implement an MSA for the prevention and control of VBDs. The guidance document presents a conceptual framework, which includes a step-by-step coordination pathway to support planning and implementation as well as sector-specific guidance for key sectors. The guidance’s main target audience includes decision makers of relevant sectors but also public health stakeholders and researchers involved in multisectoral actions for health.

### The BET Conceptual Framework

**Figure 2.**
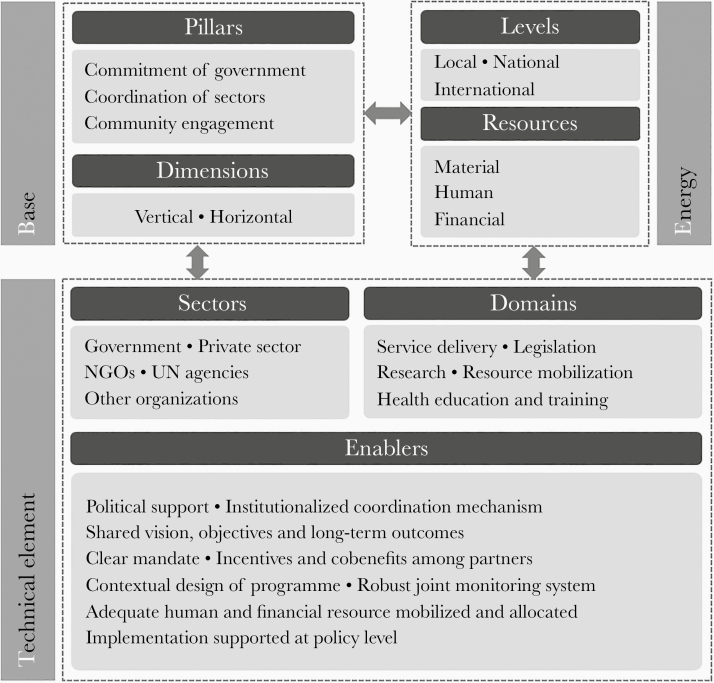
The BET (base-energy-technical) conceptual framework for multisectoral approaches to the prevention and control of vector-borne diseases. Abbreviation: NGO, nongovernmental organization. Adapted from the guidance document [13].

The conceptual framework called BET (base, energy, and technical elements) is assembled by building blocks that together encompass all the essential elements of a successful MSA (Figure 2): (1) the fundamental “3C” pillars (commitment of government, coordination among sectors, and community engagement); (2) the collaboration dimensions (vertical between levels of a hierarchical structure and horizontal among ministries as well as stakeholder groups); (3) the levels of core coordination of the MSA from local to international; (4) the resources to mobilize and share; (5) the sectors to engage, where sectors include both disciplines such as health, environment, and economy, and stakeholder groups such as government, civil society, and private sector; (6) the domains of work to be conducted through MSAs; and (7) the enabling factors. The framework was developed based on evidences, paths, results, and lessons learnt from specific cases studies and experiences in VBD prevention and control programs in which multiple sectors were involved. Other multisectoral strategies and concepts, including those mentioned above, as well as WHO’s Health in All Policies (HiAP) framework [19], were also thoroughly consulted to develop the framework which emphasizes “a facilitating, inclusive, collaborative, participatory, and sustainable mechanism” [13].

### Coordination

Effective coordination is key to success in an MSA and thus one of the fundamental 3C pillars. A coordination pathway is proposed to be coupled with the BET framework. The pathway includes 6 interrelated steps that form an iterative cycle to effectively and systematically coordinate an MSA: (1) mandate the coordination committee; (2) conduct national or local multisectoral VBDs control needs and capacities assessment; (3) build partnership; (4) sensitize the public and build capacity in all partners; (5) manage collaboration; and (6) monitor and evaluate the impact.

When countries plan to implement an MSA for the prevention and control of VBDs, it is importance to seek synergy with existing relevant programs and structures. Some multisectoral collaboration mechanisms may be available within the country for other health and nonhealth programs, such as the coordination mechanisms established for integrated vector management [20], multisectoral committee for HiAP [19], Health and Environment Linkage Initiative [21], Libreville Declaration [22], Sustainable Development Goals, and One Health Approach. With the diversity of approaches, strategies, programs, and alliances, a strong coherence between them is essential to optimize resource use and maximize impact. Examples of existing national multisectoral coordination committees include: Tanzania’s interministerial High Level Steering Committee on Nutrition [23]; Vietnam’s multidisciplinary National Traffic Safety Committee [24]; South Africa’s Technical Inter-Governmental Committee and Inter-Governmental Forum [25]; Kenya’s Inter-Agency Coordinating Committees for various health topics such as tuberculosis, malaria, nutrition, water sanitation and hygiene, and human immunodeficiency virus (HIV), as well as for health care financing [26–29].

When planning an MSA, one must secure a sufficient share of government health spending and be aware of budgets and mechanisms for the funding flows of each stakeholder. Importantly, more and better should be done for less, and the efficiency and effectiveness of funds improved. Moreover, sustainable implementation of multisectoral interventions is reinforced by appropriate regulation and legislation.

### Evidence Based on Case Studies

The main goals of MSAs for VBD control are to support countries to establish a robust mechanism that can facilitate the implementation of interventions and potentially incubate innovative strategies. However, the uptake and implementation of MSAs in full is often limited because of knowledge gaps in many areas, including the weak understanding on how to carry it out. Therefore, field testing and implementation research are required to systematically document evidence in those areas, including but not limited to the role and contribution of the different sectors and how they are working together in VBD prevention and control programs, the sectoral costs and return, and the contextual assessment of the best processes. In the same way, research is required to test the conceptual framework, the coordination pathway, and the sector-specific guidance to learn and identify the classical SWOT elements (strengths, weaknesses, opportunities, and threats), as well as areas to improve. While the guidance document [13] provides a general framework for all VBDs, without special consideration of the local contexts, the implementors need to design their contextual MSAs and programs according to needs, priorities, and specific local situations, adapting from the guidance. Research projects will be included in the second phase of this activity in TDR to test the theoretical framework through case studies. These research projects will be performed in real-life VBD control programs, with program plans developed or refined according to the guidance, and indicators designed to measure the performance. Decision makers and programs managers will be engaged in the very early stage of the projects, that is in defining the research question, and will remain participants of the field-testing process in the specific context during the research activity, with the aim to provide evidence and practical information to address knowledge gaps. The cases studies will also take advantage of the guidelines previously published [30, 31] on implementation and operational research, building multidisciplinary research teams and designing research activities.

The specific objectives of the research projects for testing the MSA framework will include:

1. Validating the guidance document [13] to identify what works and what needs to be refined;
2. Assessing the operational feasibility and implementation barriers/constraints; the attitude/incentives of the health sector to work with nonhealth sectors and that of nonhealth sectors to participate in VBD programs;
3. Identifying Specific, Measurable, Achievable, Relevant, Time-bound (SMART) indicators to measure the acceptability, efficiency, and cost-effectiveness of the programs and the overall MSA coordination system, as well as the impact of the MSA programs;
4. Identifying the pathway/mechanism that best suits the local settings and context, including how to efficiently and sustainably engage with sectors and stakeholders for specific diseases; each sector’s roles and responsibilities; as well as resource mobilization mechanisms;
5. Providing information to decision and policy makers.

Indirectly, implementation research will also help to build the capacity of the health and nonhealth workforce in the implementation of MSA programs, including the capacity related to MSA coordination. The experimental studies will serve as real-life examples that, if successful, can potentially be scaled-up and expanded to other areas. Lessons will be learned for future work.

### Capacity Building

Field testing of MSAs will provide an opportunity to strengthen the capacity of the different sectors involved in the implementation of multisectoral programs in coordination and complementarity. The capacity building will be achieved through: (1) extending understanding of VBDs determinants and their links with all relevant sectors in the local settings; (2) identifying local VBD prevention and control bottlenecks and the needs for reinforcement; (3) conducting high-quality interdisciplinary research on topics related to implementation of VBD prevention and control measures; (4) translating evidence into actions; (5) boosting willingness and capacity to collaborate, seeking synergy among sectors, and creating an enabling environment for partnership building; and (5) empowering and engaging communities.

### Monitoring and Evaluation

Monitoring and evaluation is discussed briefly in the guidance document [13]. Monitoring and evaluation has several functions and benefits. The first function is to evaluate the actual status of the implementation of a program and the activities against the planning and the targets. The second function is to identify strengths and weakness of programs, including program design, coordination mechanism, resources allocation, and so on. In addition, it can produce data for use in reducing the health equity gap, by ensuring that monitoring is disaggregated according to socioeconomic, cultural, demographic factors, including gender. Lastly, a continuous monitoring and evaluation process helps galvanize a program’s momentum. In MSAs, the system will be implemented globally for the overall process, results, and impacts. However, having sector-specific processes would be a plus. This will not only increase accountability but also facilitate information and data collection and sharing. Further, and to optimize resources, the monitoring and evaluation system should take into consideration existing sectoral and multisectoral activities and integrate whenever possible. Academic institutions and research centers are vital partners to ensure the quality of data collection, analysis, presentation, and dissemination. Different indicators should reflect both sectoral and multisectoral performances, including consideration on input, process, output, outcome, and impact (short term, intermediate, and long term). As examples, input indicators can include the amount of sectoral/total resources allocated to the MSA programs as well as obtaining a letter of endorsement from high-level government; process indicators can be the number of nonhealth sectors included, the categories of persons (stakeholder, scientists, private, and others) participating to the coordination meetings, and the level of integration of each sector at different stages estimated by scores or regression techniques. The monitoring of output, outcome, and impact should also include both sectoral and joint indicators, while impact related to each sector’s core interest should also be evaluated apart from those related to VBDs. Due to the previous lack of a systematic way of carrying out MSAs and the need to validate WHO’s new framework, more evidence on factors of success must be collected during the testing and learning process to design a comprehensive and accountable monitoring and evaluation system, which will be included in the update of the MSA guidance document [13]. Similar to the country- and context-specific design of MSA pathways, monitoring and evaluation systems should also be tailored to best determine progress and overall performance of MSAs in different settings and environments. The information resulting from monitoring and evaluation will be used by the implementors and program managers to take adaptive measures and to modify the program adequately. Therefore, as the primary users, the decision makers have to be involved in the key processes. Several tools and guidance documents have been developed on both conducting multisectoral monitoring and evaluation [32] and monitoring and evaluation of multisectoral collaborations [33, 34], in which lessons can be learned for planning and implementing MSAs for VBD prevention and control.

## CONCLUSION

In our continuous changing world, thanks to the intensive effort during the past several decades, the burden of some VBDs such as malaria has been alleviated in certain areas. Nevertheless, human health still faces increasing threats from other VBDs, such as dengue and other arboviral diseases. To properly fight these new threats, there is a need for comprehensive, robust, clearly mandated multisectoral mechanisms. A global call on MSAs with comprehensive guidance will create an important momentum in current and future VBD programs to break down the silos and empower all VBD-related and other health outcomes-related sectors and stakeholders to work together. The full deployment of robust MSAs for the prevention and control of VBDs will require a long-term and tremendous effort, but once we start to work together across different sectors, with our multidisciplinary knowledge base, concerted effort, and guiding tools available, we will be able to achieve our long-term goals.

## Notes

***Acknowledgments.*** We thank the Swiss Agency for Development and Cooperation, the International Development Research Centre, and the Swiss Tropical and Public Health Institute for their support in the first steps of this activity.

***Financial support.*** This work was supported by UNICEF/UNDP/World Bank/WHO Special Programme for Research and Training in Tropical Diseases.

***Supplement sponsorship.*** This Supplement is sponsored by TDR: the Special Programme for Research and Training in Tropical Diseases co-sponsored by the United Nations Children’s Fund (UNICEF), the United Nations Development Programme (UNDP), the World Bank and the World Health Organization (WHO).

***Potential conflicts of interest.*** All authors: No reported conflicts of interest. Both authors have submitted the ICMJE Form for Disclosure of Potential Conflicts of Interest. Conflicts that the editors consider relevant to the content of the manuscript have been disclosed.
